# Supplementary material for: Protein energy wasting in pre-dialysis chronic kidney disease patients in Benin City, Nigeria: A cross-sectional study
Source: PLoS One. 2023 May 23;18(5):e0286075. doi: 10.1371/journal.pone.0286075 (PMC10204984; doi:10.1371/journal.pone.0286075)
Supplement: Attachment — Submitted filename: Responses to Reviewer.docx [file pone.0286075.s004.docx]

Response

Comment: Kindly correct the 95% confidence interval in the abstract. "On multiple logistic regression, the factors associated with PEW in CKD were
middle aged (adjusted odds ratio: 12.50; confidence interval: 3.42-4.50; p <0.001).........." and re-submit.

Response: This been corrected and changed to (adjusted odds ratio: 12.50; confidence interval: 3.42 - 45.0; p <0.001)

Comment: Please clarify the sources of funding (financial or material support) for your study. List the grants or organizations that supported your study, including funding received from your institution.

Response: This study was self-funded by the authors. No financial or material support was received from any organization for this work

Comments: State what role the funders took in the study. If the funders had no role in your study, please state: “The funders had no role in study design, data collection and analysis, decision to publish, or preparation of the manuscript.”

Response: The authors received no specific funding for this work.

Comments: If any authors received a salary from any of your funders, please state which authors and which funders.

Response: The authors received no specific funding for this work.
